# Synovial Fluid White Cell Count in Knee Osteoarthritis: Association with Structural Findings and Treatment Response

**DOI:** 10.1002/art.39829

**Published:** 2017-01

**Authors:** Paul S McCabe, Matthew J Parkes, Nasimah Maricar, Charles E Hutchinson, Anthony Freemont, Terence W O’Neill, David T Felson

**Affiliations:** 1 Faculty of Medical and Human Sciences, Arthritis Research UK Centre for Epidemiology, Institute of Inflammation and Repair, Manchester Academic Health Science Centre, University of Manchester, Manchester, UK; 2 NIHR Manchester Musculoskeletal Biomedical Research Unit, Central Manchester NHS Foundation Trust, Manchester Academic Health Science Centre, Manchester, UK; 3 Warwick Medical School, The University of Warwick, Coventry, UK; 4 MRC/EPSRC Molecular Pathology Node and Centre for Regenerative Medicine, Institute of Inflammation and Repair, Faculty of Medical and Human Sciences, The University of Manchester, Manchester, United Kingdom; 5 Department of Rheumatology, Salford Royal NHS Foundation Trust, Salford, UK; 6 Clinical Epidemiology Unit, Boston University School of Medicine, Boston, Massachusetts, USA

## Abstract

**Objectives:**

Osteoarthritis (OA) is a disease with a significant inflammatory component. The aim of this analysis was to determine the relationship between synovial fluid white cell count (SF WCC) and two parameters: disease severity and the reduction in knee pain after intra-articular steroid injection.

**Methods:**

Subjects with painful knee OA were recruited for participation in an open label study of intra-articular steroid therapy. Information was obtained about knee pain using the KOOS questionnaire and a proportion of subjects had magnetic resonance imaging (MRI) performed. Prior to injection with methylprednisolone acetate (80mg), the index knee joint was aspirated and the fluid obtained forwarded for assessment of SF WCC.

**Results:**

Information on SF WCC was available in 55 subjects. An increase in white cell count category (< 100, 101–250 and > 250–1,000 cells/mm^3^) was associated with an increase in synovial tissue volume (p = 0.028) and with other MRI-based measures of disease severity. Also, with each category increase in SF WCC there was a greater mean reduction in KOOS pain score after steroid injection; ≤100 cells/mm3 12.5 (SD 19.9) [referent], 101–250 cells/mm^3^ 21.3 (SD 20.6) [β coefficient 0.279 p=0.049 ] and 251–1000 cells/mm^3^ 29.3 (SD 15.2) [β coefficient 0.320 p=0.024].

**Conclusion:**

Although within the ‘normal’ range, total synovial fluid white cell count appears to be a biomarker for MRI synovitis and may also predict response to anti-inflammatory treatment.

Osteoarthritis (OA) is increasingly recognised as a disease with a significant inflammatory component.[1] Recent studies suggest that up to 90% of subjects with knee OA have evidence of synovial thickening on magnetic resonance imaging (MRI).[2] Synovial thickening on MRI has been shown in persons with symptomatic knee OA to be correlated with macroscopic scoring of synovitis (r=0.61) and histologically with infiltration of inflammatory cells into the subsurface layers of synovium (r=0.54).[3] We have shown recently in symptomatic knee OA that there is a correlation between synovial tissue volume and pain severity.[4]

Synovial fluid (SF) white cell count (WCC) has long been recognised to have utility in assessment and diagnosis of arthritis.[5,6] However SF, WCC in osteoarthritis is typically designated as a non-inflammatory fluid (defined by Freemont as <1000 cells/mm^3^)[7] without any further stratification within that range.

Intra-articular steroid injections are a frequently used symptomatic treatment in knee OA but in clinical practice the response to therapy is mixed; some patients experience dramatic improvement in pain post injection whilst others derive little, if any, benefit. A wide variety of putative predictors of response to IA steroids have been evaluated previously though none have been found to be reliable predictors of treatment response (reviewed in [8]). Surprisingly, particularly given the frequency with which SF is obtained in research and clinical practice, the role of SF WCC as a potential predictor of treatment response or a marker of disease activity has not been fully evaluated.

We hypothesised that higher SF WCC levels within the non-inflammatory range, as a marker of low grade inflammation would be associated with increased synovial tissue volume and pain and might identify persons more likely to respond to an anti-inflammatory treatment. Using data from a recent open label trial of intra-articular corticosteroid injection (IACI).[4] The aim of this study was to determine the clinical and structural correlates of SF WCC in those with symptomatic knee OA.

## MATERIALS and METHODS

120 men and women aged ≥ 40 years were recruited for participation in an open label study assessing the efficacy of IACI in knee OA and including assessment of structural change by MRI (ISRCTN: 07329370). Eligibility including inclusion and exclusion criteria have been published previously.[4] We studied one knee per participant with a requirement that subjects had at least moderate knee pain for 48 hours in the preceding 2 weeks or scored >7 on Knee Injury and Osteoarthritis Outcome Score (KOOS)[9] questions P2-P9. A further 80 participated in an extension study which, because of costs, did not utilise MRI imaging but did not otherwise differ. Participants were required to have painful primary knee OA evidenced by radiographic, MRI or arthroscopy changes (Kellgren-Lawrence [K-L] score ≥2 or typical changes of OA with at least cartilage loss). At baseline participants completed questionnaires including the KOOS, the pre-planned primary pain outcome and visual analogue scale (VAS) score for pain during a nominated activity the participant found most painful (VAS_NA_). Participants in the main, though not the extension, study had a contrast enhanced MRI (CE-MRI) scan performed followed by arthrocentesis and injection of 80mg methylprednisolone acetate without local anaesthetic and were advised to rest for a day after the injection. Subjects were seen again usually within 2 weeks at which time they underwent repeat assessment including completion of KOOS and VAS_NA_ and had another CE-MRI. Participants provided written informed consent and this study was approved by the Leicestershire multicentre research ethics committee (reference 09/H0402/107).

### Synovial fluid analysis

During arthrocentesis, SF, if present, was withdrawn and decanted into a 2ml lithium heparin tube. Those not immediately transferred to the laboratory were stored below 5°C and analysis was performed within 48 hours. Samples were processed in an experienced laboratory which provided a regional SF analysis service. Analysis was performed using the method described by Denton[10] with WCC determined using a Fuchs-Rosenthal counting chamber following staining with 0.01% w/v methyl violet 6b in normal saline or using an automated Cellometer Vision (PEQLAB Ltd, Sarisbury Green, UK) following staining with 0.002% w/v acridine orange *s*olution depending on equipment availability. Participants with a SF WCC >1500 cells/mm^3^ (n=2) were withdrawn from the trial due to concerns they might have primary inflammatory arthritis.

### Magnetic resonance imaging assessment

At baseline and follow up participants in the MRI study underwent a CE-MRI of the knee performed using a 3T Phillips MRI. STV was determined by manual segmentation of the synovial tissue layer on the sagittal post-contrast T1W FS image by a single observer followed by computer image analysis as described previously.[4] The ICC for synovial volume was 0.94 (p<.001).[4] A sub-sample (N=101 in the MRI study) also underwent semi-quantitative assessment of cartilage (14 areas scored 0–6, maximum score 84), osteophytes (14 areas scored 0–7, maximum score 98) and BMLs (15 areas scored 0–3, maximum score 45) by an experienced radiologist using the Whole-Organ Magnetic Resonance Imaging Score (WORMS).[2] Semi-quantitative assessment of synovitis was performed at 7 regions, each scored (0–3) as described by Roemer *et al*[2]. Intra-reader reliability assessment was assessed by the same reader re-evaluating 19 films after an interval period with weighted kappas of 0.63 to 0.88 depending on the feature.

### Statistical analysis

Participants were divided into tertiles based on SF WCC; those with ≤100 cells/mm^3^, 101–250 cells/mm^3^ and 251–1000 cells/mm^3^. At baseline, depending on the distribution of the variable, we used parametric (ANOVA) or non-parametric (Kruskal-Wallis) tests to determine the significance of differences in symptoms and structural change between groups. χ^2^ were used to test the differences in categorical data. We used linear regression to assess the association between SF WCC and within person change in pain and synovial tissue volume. Statistical analysis was undertaken using Stata V.13.1 (StataCorp, College Station, Texas).

## RESULTS

### Subject characteristics

A SF aspirate was obtained from 93 participants of whom 67 had SF analysis performed. Of these the median volume of synovial fluid was 4ml (IQR 2–9ml). Compared to those who had synovial fluid analysis, those who did not were not significantly different in terms of their age (66.6 years vs 62.6 years), and pain at baseline (KOOS : 44.2 vs 45.7 ; NA-VAS 6.8 vs 7.0). The laboratory reported a precise SF WCC for 47 samples (70.1% of all SF analysed) with the remainder reported as either ‘<100’ (N=8, 11.9%) or ‘<500’ (N=12, 17.9%) cells/mm^3^. Due to the uncertainty about the WCC estimate; those reported as ‘<500 cells/mm^3^’ were excluded from further analysis, leaving 55 subjects; 37 with MRI data and 18 without. The mean age of those 55 subjects with data on SF was 61 years (SD=10.3) and just under two thirds were men. The majority of participants had a K-L grade of 2 or 3. Mean KOOS pain at baseline was 46.6 (SD=15.6), see Table 1.

### Association between SF WCC, knee pain and structural parameters

We found no significant association between SF WCC and either age, pain at baseline as assessed by KOOS or VAS_NA_, or with disease severity as assessed by K-L score. In contrast there was a significant increase in synovial tissue volume with increasing WCC category; also an increase in whole knee semi-quantitative scoring of synovitis, cartilage loss, and BML score with increasing WCC category. There was, however, no association with osteophyte score, see Table 2.

### SF WCC and change in pain and structure following steroid injection

Subjects were reviewed a median of 8 days (interquartile range 8 to 14 days) following baseline assessment. Compared to those with a WCC < 100 cells/mm^3^ there was a greater within person reduction in KOOS score in those with a WCC between 101–250 cells/mm^3^ (β coefficient 0.279, p=0.049) and 251–1000 cells/mm^3^ (β coefficient 0.320, p=0.024), see Table 3. Further adjustment for the amount of synovial fluid removed did not affect the significance of the results. A similar trend of greater pain reduction with increasing synovial fluid WCC was observed for VAS_NA_ but this did not reach statistical significance (≤100 cells/mm^3^ referent; 101–250 cells/mm^3^, β coefficient -0.242, p=0.107; 251–1000 cells/mm^3^, β coefficient -0.272, p=0.072). Increasing WCC was associated with a greater within person change in synovitis as measured both quantitatively and semi-quantitatively though the association did not attain statistical significance.

## DISCUSSION

This is the first study to assess the association between SF WCC and knee joint structure assessed by MRI in knee OA. Even though all participants in the analysis had SF WCC within the ‘normal’ range, our data showed that increasing SF WCC was correlated with more severe knee OA and also synovitis as defined by synovial tissue volume. Additionally those with higher SF WCC at baseline experienced greater reduction in knee pain following an intra-articular steroid injection.

Our finding of an association between SF WCC and synovitis at baseline assessed either quantitatively or semi-quantitatively is consistent with findings of an increased SF WCC and other measures of inflammation in primary inflammatory arthritides. [5,6,10,11] We found no association of SF WCC with K-L grade though the numbers of those with more severe disease (N=3 for K-L 4) was small. To our knowledge there are only two other studies that have looked at the association between SF WCC and K-L grade; neither showed evidence of any association [12,13].

We also identified an association between increasing baseline SF WCC and the reduction in pain assessed by KOOS score following intra-articular steroid injection. Those is the highest tertile of SF WCC experienced a greater than two fold greater reduction in pain assessed by either KOOS or VAS_NA_ compared to those in the lowest. These findings suggest that SF WCC could have a role in predicting treatment response. This or other measures of synovial inflammation may even facilitate treatment stratification. One previous study reported no association between baseline WCC and change in VAS pain following intra-articular steroid injection in knee OA, though only 16 subjects were studied .[13]

There are a number of limitations which need to be considered in interpreting our data. This study was a post-hoc analysis using existing data and the number of participants included in this analysis is small (N=55 with 37 contributing MRI data). Some caution is therefore required in interpretation. Our primary rationale for assessing SF WCC was to identify and exclude participants with an alternate diagnosis. Therefore during the conduct of this study we accepted reporting of SF WCC as either ‘<100’ or ‘<500’ cell/mm^3^ as these were below the exclusion criteria cut off. However, the manner in which SF WCC was reported led to a reduction in our sample size, due to the exclusion of from participants with WCC reported as ‘<500’ cells/mm^3^, and prevented analysis using SF WCC as a continuous variable. Both of these limitations served to reduce the power of the study. Further studies with larger number of participants are required to confirm the findings.

The clinical utility of our findings remains uncertain. It is possible that a high SF WCC within the normal range may identify persons more likely to benefit from an intra-articular steroid injection and even from other treatments targeted to joint inflammation. Our results highlight the need for further research to assess the potential value of SF WCC in OA. SF WCC may be an overlooked, yet easily measurable,[14] marker of OA disease severity or even a predictor of treatment response. These findings may important implication in both the design of future research studies and clinical practice.

## Figures and Tables

**Table 1:** Baseline characteristics for all subjects

Variable	Statistic

	Mean (SD)
**Age (years)**	61.0 (10.3)
**Gender**	**N (%)**
Male	36 (65.5)
Female	19 (34.6)
**Maximal Kellgren-Lawerence score in either** **patellofemoral or tibiofemoral compartment**	**N (%)**
2	19 (37.3)
3	29 (56.9)
4	3 (5.9)
**Pain**	**Mean (SD)**
KOOS pain*	46.6 (15.6)
Nominated activity VAS*	7.0 (1.8)
**MRI Volumetric Assessment**	**Median (IQR)**
Synovial tissue volume (mm^3^)	9667 (6320–13231)
**Whole knee semi-quantitative assessment**	
Synovitis†	15 (12–16)
Bone marrow lesion‡	6 (3–9)
Cartilage‡	11.5 (6–18)
Osteophytes ‡	36 (20–53)

* KOOS scored from 100 (no pain) to 0 (extreme pain), Nominated activity VAS scored from 0 (no pain) - 10 (extreme pain). Abbreviations: KOOS - Knee Injury and Osteoarthritis Outcome score,

† Sum of 7 regions (each scored 0–3)

‡ Assessed using the Whole Organ Magnetic Imaging Score. Whole knee scores calculated as the sum of individual compartment scores

**Table 2:** Association between synovial fluid white cell count and clinical and imaging parameters at baseline

	Synovial fluid white cell count (cells/mm^3^)			
Variable	≤100	101–250	251–1000	

	Mean (SD)	Mean (SD)	Mean (SD)	p
**Age**	61.1 (10.7)	62.2 (10.8)	58.7 (8.9)	0.635
**Gender**	**N (%)**	**N (%)**	**N (%)**	
Male	15 (71.4)	15 (68.2)	6 (50.0)	
Female	6 (28.6)	7 (31.8)	6 (50.0)	0.434
**Maximal Kellgren-Lawerence score in either** **patellofemoral or tibiofemoral compartment**				
2	8 (38.1)	7 (33.3)	4 (44.4)	
3	12 (57.1)	12 (57.1)	5 (55.6)	
4	1 (4.8)	2 (9.5)	0 (0)	0.867
**Baseline Pain**				
KOOS pain	46.3 (16.4)	49.5 (17.0)	41.9 (11.0)	0.405
Nominated VAS	7.0 (1.7)	6.6 (2.2)	7.6 (1.1)	0.346
**MRI Volumetric Assessment**	**Median (IQR)**	**Median (IQR)**	**Median (IQR)**	
Synovial tissue volume (mm^3^)	7771 (6082–9667)	10670 (6036–13154)	13934 (7441–20333)	0.028
**Whole knee semi-quantitative assessment**				
Synovitis †	10.5 (8–13)	13 (11–14)	15 (13 – 15.5)	0.016
Bone marrow lesion‡	3 (1.5–6.5)	5 (4–7)	8 (7–10.5)	0.013
Cartilage‡	5 (2–10.5)	11.5 (9–16)	18 (13.75–19.5)	0.003
Osteophytes‡	20.5 (17–43.5)	43 (31–53)	38.5 (29–49.5)	0.170

KOOS scored from 100 (no pain) to 0 (extreme pain), Nominated activity VAS scored from 0 (no pain) - 10 (extreme pain),. Abbreviations: KOOS - Knee Injury and Osteoarthritis Outcome score.

† Sum of 7 regions (each scored 0–3)

‡ Assessed using the Whole Organ Magnetic Imaging Score. Whole knee scores calculated as the sum of individual compartment scores

**Table 3:** Association between synovial fluid white cell count and within person change in pain between baseline and follow up

Variable	Synovial fluid white cell count (cells/mm^3^)	Mean within person change in pain (SD)	Unstandardised coefficient	95% CI	Β*	p
**Pain**						
KOOS pain	**≤100**	12.5 (15.2)	referent			
	**101–250**	21.3 (20.6)	11.1	(0.03 – 22.17)	0.279	0.049
	**251–1000**	29.3 (15.2)	15.07	(2.06 – 28.09)	0.320	0.024
Nominated VAS	**≤100**	−1.9 (2.3)	referent			
	**101–250**	−3.1 (2.8)	−1.4	(−3.12 – 0.32)	−0.242	0.107
	**251–1000**	−4.0 (3.2)	−1.82	(−3.81 – 0.17)	−0.272	0.072

Results of linear regression.

Abbreviations: β*- standardised beta coefficient. KOOS - Knee Injury and Osteoarthritis Outcome score
